# Hunting for an Antigen: Humoral Immunity in Alzheimer’s Disease

**DOI:** 10.3390/cells15131227

**Published:** 2026-07-07

**Authors:** Angel M. Delgado, Braxton D. Greer, Robert W. Maul, Patricia J. Gearhart

**Affiliations:** 1 Laboratory of Molecular Biology and Immunology, National Institute on Aging, National Institutes of Health, Baltimore, MD 21224, USA; angel.delgado@nih.gov (A.M.D.); braxton.greer@nih.gov (B.D.G.); maulrw@mail.nih.gov (R.W.M.); 2 Graduate Program in Immunology, Johns Hopkins University School of Medicine, Baltimore, MD 21205, USA

**Keywords:** Alzheimer’s disease, inflammation, B cells, neurodegeneration

## Abstract

**Highlights:**

**What are the main findings?**

- B cell involvement in Alzheimer’s disease is unclear, with evidence suggesting both protective and pathogenic capacities, resulting in direct and inverse relationships with disease severity.
- The presence of B cells and immunoglobulin in the central nervous system implies that an immune reaction is occurring. However, it is uncertain whether antibodies are directed against amyloid and tau; thus, the search for antigens continues.

**What are the implications of the main findings?**

- Rigorous characterization of antibody-secreting cells and immunoglobulin will contribute to further understanding adaptive immune dynamics in Alzheimer’s disease.
- Identification of antigen targets to known (amyloid-beta and tau) or novel antigens will contribute to clinical immunotherapeutic development.

**Abstract:**

Alzheimer’s disease (AD) is the leading cause of dementia, affecting millions of individuals on a global scale. A fatal and incurable neurodegenerative disease, AD is defined by various molecular and cellular abnormalities, such as the formation of intracellular neurofibrillary tangles and extracellular amyloid plaque deposition, leading to increased neuroinflammation, parenchymal tissue breakdown, and cognitive deficiencies. These pathological conditions are associated with the disruption of the blood–brain barrier (BBB), which is the protective network of cells responsible for maintaining homeostasis at the borders of the central nervous system (CNS). The breakdown of the BBB results in a dysregulation of the neuroimmune axis. The induction of inflammatory and autoimmune responses has been a key topic of study in AD, particularly surrounding innate immune cell activation. Recent discoveries focusing on the adaptive immune branch in the diseased CNS show evidence of effector and memory T cell activation and expansion, highlighting the complex relationship of the neuroimmune axis. It is speculated that humoral immunity might play a significant role in pathology through the production of autoantibodies. However, the contribution of B cells and plasma cells is unclear. We aim to review the literature addressing the following questions: are B cells protective or pathogenic in the CNS during AD, and do their antibodies have specific antigenic targets within this niche? The characterization of humoral contributions to immune dysregulation in AD is critical to the development of novel therapeutic strategies to slow or prevent neurodegeneration and cognitive impairment.

## 1. Introduction

Alzheimer’s disease (AD) is one of the most predominant neurodegenerative pathologies in the world and is the leading cause of dementia in aged individuals [1]. In the United States alone, approximately 6 million people are clinically diagnosed with AD, with the number predicted to double to 13 million by 2050 [2,3]. Globally, an estimated 50 million individuals are afflicted with dementia, with the number to triple in the next 30 years [4,5]. Assessments calculating lifetime risk for Alzheimer’s have been shown to be around 10% in men and 20% in women [3,6]. With elevated predisposition in older individuals coupled with an ever-increasing aging population, AD is a significant public health problem: a complex and fatal disease to which no known cure has yet to be identified; more basic and clinical research efforts are underway, due to how quickly cases seem to be on the rise.

**Figure 1:**
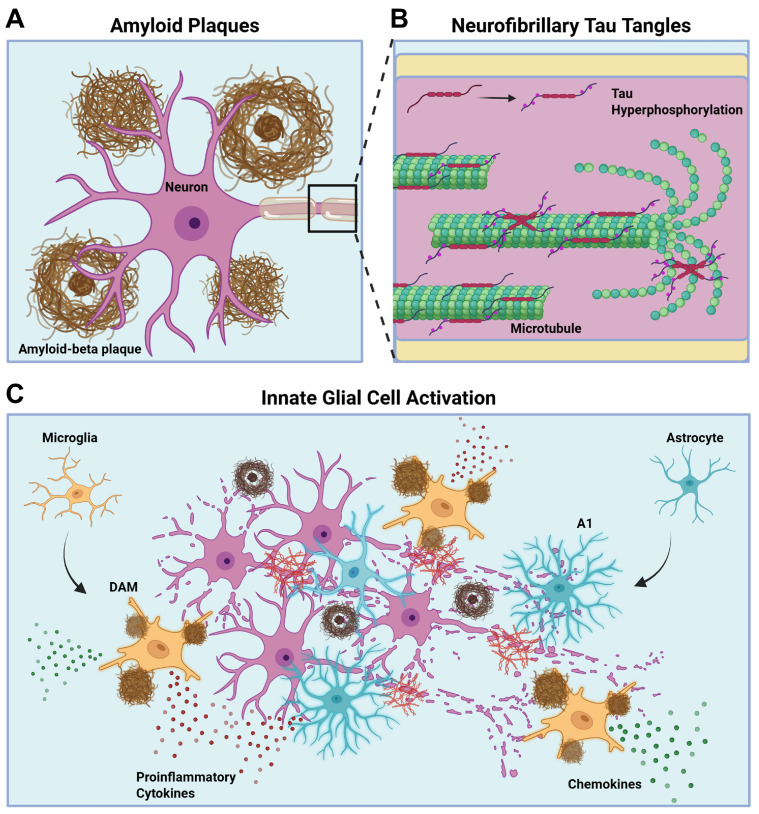
Schematic representation of changes during AD. (**A**) Amyloid plaques. Accumulation of extracellular amyloid-beta aggregates form insoluble fibrous plaques, as visualized in neuronal axons (black box). (**B**) Neurofibrillary tau tangles. Hyperphosphorylation of tau stabilizing protein and formation of intracellular neurofibrillary tangles results in microtubule dysfunction. (**C**) Innate glial cell activation. Amyloid plaque internalization activates innate glial cells (microglia and astrocytes) into proinflammatory states (DAM and A1, respectively). Elevated glial-derived cytokine and chemokine production results in neuronal breakdown. Created in BioRender. Maul, R. (2026) https://BioRender.com/jly9egg.

AD is defined by two abnormalities in the brain at the molecular level: the accumulation of extracellular amyloid-beta plaques and the formation of intracellular neurofibrillary tangles of tau protein [7] (Figure 1A,B). For amyloid-beta protein (Figure 1A), mis-regulation of its metabolism and clearance are the central points for plaque pathology. Amyloid precursor protein (APP) is a single-pass transmembrane protein which is processed into soluble subunits, diverging into either an anti-amyloidogenic or amyloidogenic pathway, depending on which enzymes are recruited. For the anti-amyloidogenic side, APP is cleaved in the middle of its partially intramembrane amyloid-beta region by α-secretase, releasing the N-terminal ectodomain (sAPP-α) and leaving behind the membrane-bound C-terminal fragment C83 (αAPP). The intramembrane portion of C83 is then targeted by γ-secretases, thus releasing a free extracellular (p3) and intracellular signaling peptide (AICD) [8,9]. However, the amyloidogenic pathway differs in that the initial proteolytic process is mediated instead by β-secretase, also known as beta-site amyloid precursor protein-cleaving enzyme 1 (BACE-1). BACE-1 cleaves the amyloid-beta region of APP closer to the N-terminal side, producing a shorter secretory fragment (sAPP-β) and a longer transmembrane C-terminal fragment C99. The secondary cleavage event is mediated by γ-secretases, still producing AICD, although the newly processed product, amyloid-beta, is released into the extracellular environment [8,9]. Amyloid-beta peptides are produced in variable lengths, of which some have a higher potential to aggregate into smaller oligomeric forms (Ab40 and Ab42) or larger fibril-like secondary structures, resulting in both soluble and insoluble plaques, respectively. The leading hypothesis is that this phenomenon directly contributes to the AD phenotype.

The second abnormality occurs with the induction of neurofibrillary tangles consisting of aggregated hyperphosphorylated tau [10] (Figure 1B). Tau is a microtubule-associated protein largely found in the axon that maintains neuronal cytoskeletal stability and normal vesicle transport capability. Tau functionality can be affected by post-translational modifications and is hyperphosphorylated in neurological diseases, with abnormally modified tau containing around 2–4 fold more phosphate groups than under normal conditions [11,12]. This hyperphosphorylation of tau causes a shift in binding affinity, resulting in its detachment from microtubules and self-aggregation into oligomeric fibrils known as paired helical fragments. These subsequently comprise larger neurofibrillary tangles and result in microtubule disassembly and synaptic dysfunction [8,12,13,14]. The co-formation of amyloid plaques and tau protein tangles in aged tissue has been repeatedly shown to be neurotoxic, driving increased neurodegeneration and severe cognitive deficiency [15,16,17,18,19].

The identification and accurate measurement of these AD biomarkers (amyloid-beta and tau) have been crucial for the evolution of neurodegenerative diagnostics. In the early 2010s, a restructuring was initiated for how AD was clinically defined, with molecular characteristics being heavily emphasized as a requirement [20]. Cerebrospinal fluid (CSF) has historically been used as the predominant sample for pre-mortem AD biomarker detection, and studies have shown amyloid-beta and tau species are found at different levels. Amyloidosis is detected at earlier stages of the disease, termed preclinical AD. Gross tau pathology is detectable decades later, alongside canonical dementia-associated synaptic dysfunction [14,21,22,23].

Many investigative efforts sought to target these pathological biomarkers with the goal of ameliorating disease, including direct immunizations with amyloid-beta protein or indirect immunizations via transfer of anti-amyloid antibodies. Treatments in animal models were initially promising, showing diminished plaque burden and reduction in cognitive impairment [24,25,26,27,28,29,30,31]. Subsequently, the transition of these practices into humans had mixed efficacy: most had amyloid deposition reversal, but no benefit towards cognitive impairment or neuroinflammation [32,33,34,35]. More recently, two monoclonal immunotherapies were successful in showing their capacity to delay the progression of cognitive decline [36,37]. However, there are reports highlighting adverse reactions to these immunotherapies during clinical trials. A common effect across treatments is amyloid-related imaging abnormalities (ARIAs) that produce cerebral edema and hemorrhages, and in some cases even death [36,37,38]. More recent strategies targeting the amyloid or tau phenotype are proving to be increasingly safer; nonetheless, patients have not been rescued from cognitive decline [39,40,41,42]. With multiple clinical interventions being unsuccessful in preventing neurological degradation, an understanding of the key players in the dysregulated central nervous system (CNS) niche is required. To study AD pathology within a pliable context, several *in vivo* mouse models are used to investigate the effects of these abnormal protein manifestations, as will be detailed in the following section.

## 2. Mouse Models of Alzheimer’s Disease

### 2.1. Amyloid Mice

Murine models are genetically engineered to express dysregulated amyloid pathology, as summarized in Table 1. These mice possess human transgenes harboring key mutations in genes associated with the heritable form of AD, known as familial Alzheimer’s disease (FAD) [43,44,45,46,47,48]. Commonly used AD models are the APP/PS1 and 5xFAD transgenic lines, each expressing mutated human *APP* and Presenilin 1 (*PSEN1*) genes driven by the neuron-specific *Thy1* promoter, as their aggressive amyloidogenic phenotype manifests early in adulthood. The APP/PS1 mouse is a double transgenic featuring the *APP* “Swedish” (K670N/M671L) and *PSEN1* substitutions (L166P) that result in elevated total amyloid-beta production and processing into Ab42, respectively. Hippocampal plaque formation and microgliosis are shown to occur early at 3–4 months of age, with expansive deposition of large dense cores surrounded by diffuse amyloid-beta becoming apparent by 7–8 months, contributing to significant cognitive deficiency [46,49].

On the other hand, the double transgenic 5xFAD mouse expresses five FAD mutations, namely, the *APP* “Swedish”, “Florida” (I716V), and “London” (V717I) substitutions, as well as *PSEN1* mutations (M146L and L286V). Each subsequent FAD mutation has been shown to be additive in its amyloidogenic phenotype of increased Ab42 synthesis, regardless of whether the mutations reside solely in the *APP* or *PSEN* gene, or both. The 5xFAD line is an accelerated model of plaque pathology due to its rapid and heavy Ab42 burden and early elevated gliosis at 2 months of age, as well as memory impairment by 3–6 months, which is notably faster and more severe than the other models. It is also characterized by increased synaptic dysfunction and cortical neurodegeneration at 9 months [47,50].

### 2.2. Amyloid Plus Tau Mice

While the APP/PS1 and 5xFAD models are robust tools to study the amyloid branch of AD onset, a third model is often incorporated in these studies to cover the remaining molecular hallmark of tau aggregates. The triple transgenic 3xTgAD mouse is unique in the sense that it develops both amyloid plaques and neurofibrillary tau tangles. It co-expresses three FAD mutations, namely, the *APP* “Swedish” and *PSEN1* (M146V) substitutions and a mutation (P301L) in the Microtubule-Associated Protein Tau gene (*MAPT*). The *APP* and *PSEN1* mutations result in elevated Ab42 formation, while the *MAPT* mutation promotes assembly of tau-paired helical fragments. The 3xTgAD model features amyloid deposits and synaptic dysfunction in the cortex and hippocampus around 6 months of age. While cognitive defects have been detected as early as 4 months, tau pathology is not as apparent until around 12 months [48,51].

### 2.3. AD Model Limitations

This robust arsenal of AD murine models makes it possible to study the intricate molecular dysregulations among multiple cell populations within the CNS; however, there are three limitations to their use that should be addressed. The first limitation lies in the variability of the pathogenic phenotypes due to the parental origin of the transgene. Careful tracking of the breeding scheme revealed that maternal inheritance (Female-5xFAD crossed to Male-C57BL/6) leads to reduced amyloid plaque burden in the progeny, relative to their paternal inheritance counterparts (Male-5xFAD crossed to Female-C57BL/6). This is due to genomic imprinting of the *Thy1* promoter, which contains a CpG island that may be predisposed to epigenetic silencing, thus modulating protein expression of the transgenes (*APP* and *PSEN1*) [53]. While this has not been reported outside of the 5xFAD model, it is expected to be conserved in APP/PS1 and 3xTgAD mice because their transgene expression is also driven by a *Thy1* promoter. It was also verified that the genomic imprinting was still pertinent in an unrelated reporter strain (ATP biosensor line) under the same promoter [53].

The second limitation is that these transgenic models have non-endogenous levels of mutated APP, resulting in increased APP byproducts outside of amyloid-beta which may produce phenotypic artifacts. To combat this, a humanized *APP* knock-in construct is sometimes used to selectively produce more Ab42 without overexpressing APP. An example is the APP-NLGF model designed to express mutated APP at baseline levels through an endogenous promoter and humanized amyloid-beta region. This model features three FAD mutations, namely, the *APP* “Swedish”, “Iberian” (I716F), and “Arctic” (E693G) substitutions. The latter two mutations bias amyloid processing to favor the Ab42 form and facilitate oligomerization, respectively. These mice display aggressive amyloidosis, with plaque formation appearing at 2 months, memory impairment at 6 months, and elevated gliosis at around 9 months [52]. Thus, the APP-NLGF model is a favorable alternative for dealing with this limitation. It also avoids the first limitation of genomic imprinting because its construct lacks the *Thy1* promoter, unlike the 5xFAD counterpart [53].

The third limitation of these models is that they cannot fully encompass AD due to their heterogeneity. FAD, characterized by the inheritance of select autosomal dominant mutations within the *APP* and *PSEN1* genes, is a rare occurrence of less than 1% of total AD diagnoses [1,4]. The most prevalent form is spontaneous (or sporadic) AD, which accounts for more than 95% of cases. It features the same incidences of amyloidosis, but also several risk factors such as aging, genetics, and environment, which contribute to the overarching complexity of this neurodegenerative disease [1,4,54]. Nonetheless, it is too difficult to account for these numerous and unpredictable factors within an experimental model system. While the AD transgenic models are very useful and have their faults, human studies are necessary to confirm these findings, and together they are critical to understanding the components of diseased CNS. One component is the immune system, which has been a key focus in the field, yielding rewarding results.

## 3. The Neuroimmune Axis

**Figure 2:**
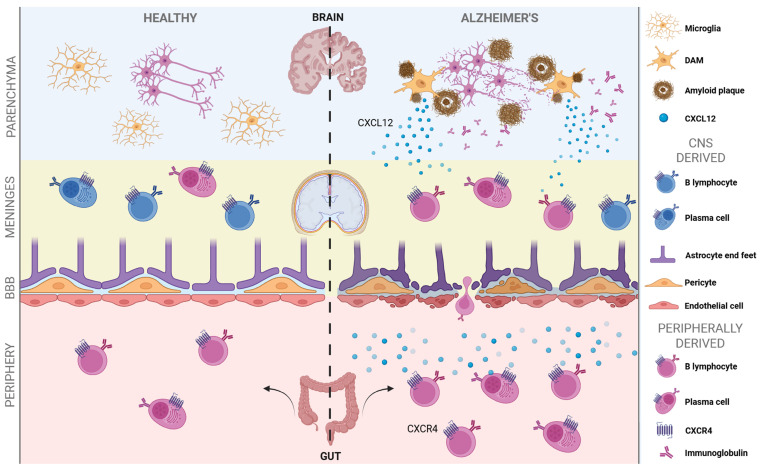
B cell and plasma cell infiltration during AD development. Under healthy conditions, CNS and gut-derived B cells and plasma cells reside in the meninges. The BBB comprises endothelial cells, pericytes, and astrocyte end-feet. During AD, chemokine CXCL12 signaling from DAMs is elevated within the parenchyma. Simultaneously, increased BBB permeability facilitates the recruitment of CXCR4-positive B cells and plasma cells from the gut to the meninges. Immunoglobulins colocalize with DAM and amyloid plaques in the parenchyma. Created in BioRender. Maul, R. (2026) https://BioRender.com/zczb5rm.

The CNS has been described historically to be immune-privileged due to the presence of selective barriers, such as the blood–brain barrier (BBB), that serve to maintain a highly regulated separation from influences of the systemic environment. The BBB consists of several layers of tight protective shielding surrounding the cerebral vasculature, comprising endothelial cells, followed by a layer of perivascular cells, all finely wrapped by a secure border of astrocyte end-feet (Figure 2; BBB). It was thought that this isolation would prevent recognition from the body’s own defense mechanism (i.e., the immune system), as it has been shown in other privileged sites that breach of this barrier results in increased inflammation and autoimmunity [55,56]. However, the recent literature more accurately describes the relationship between the CNS and the rest of the body as immune surveillance, rather than immune privilege, because of their proximal location to one another in specialized compartments where they regularly influence each other’s homeostasis directly and remotely [57,58,59].

Robust characterization of the CNS has uncovered an extensive immunological network consisting of several populations of non-glial myeloid and lymphoid-derived cells [60,61]. A key set of structures that serves as a neuroimmune interface are the meninges (Figure 2; Meninges). The meninges consist of three layers of tissues located between the skull and the brain parenchyma: the dura mater, arachnoid, and pia mater. The most superficial layer of the meningeal tissue, the dura mater, contains active myeloid-derived cells and lymphocytes critical for preserving cognitive health [62,63,64,65,66]. Further investigation revealed that these immune cells can be mobilized throughout different sections of the CNS, such as the leptomeninges (arachnoid and pia mater) and subarachnoid space. This mobility is assisted by the presence of lymphatic vessels, leading to questions regarding the origin of the cells [67,68]. The revolutionary discovery of vascular channels throughout the skull uncovered the calvaria bone marrow, a hematopoietic niche, as a source capable of supplying immune cells into the meninges, where they may traffic to the outer borders of the CNS [69,70,71,72,73].

Another notable location harboring immune residence is the choroid plexus, a specialized tissue in the ventricles responsible for CSF production [74]. CSF routes through the subarachnoid and perivascular spaces serving as a bridge for context-dependent signals (e.g., cytokines and antigens) to establish robust communication between the parenchyma and immune-occupying compartments (marrow and meninges), allowing for environmental perturbations to influence changes in homeostatic dynamics [75,76]. A hematopoietic environment that offers exposure to CNS-specific signals implies the existence of a selection mechanism for tolerance. Systemically, the adaptive immune system is educated to distinguish self from non-self to prevent the rise in autoimmune populations, though it is uncertain whether this holds true for the CNS, due to the previously inaccurate identification of the niche as an immune privileged site. As lymphocyte ontogeny is heavily responsible for this phenomenon, it is speculated that the heterogeneous populations of developing lymphocytes that are localized to the skull bone marrow and dura mater are rigorously screened for autoreactivity through exposure to CNS antigens present in the CSF. This hypothesis is feasible because of evidence showing that CSF-mediated signals can influence hematopoiesis and mobilization of CNS-resident immune cells. Specifically, the lymphocyte compartment is capable of recognizing self-antigens and undergoing negative selection locally [71,75,76,77,78]. This suggests the presence of a newfound branch of CNS-derived adaptive immunity, which serves as a pillar for protection and promotes an additional avenue of research regarding neurological immune function.

## 4. Innate Intervention by Glial Cells

Delineation of the vast interconnected network of CNS-residing immune cells is essential for understanding the immune dysregulation occurring in AD. The extracellular accumulation of amyloid-beta and tau influences the recruitment of glial cells (astrocytes and microglia) that are tasked with preserving neuronal homeostasis through structural support and the disposal of waste byproducts [79,80,81,82,83]. The internalization of amyloid and tau aggregates drives glial activation and transition into chronic-reactive states (e.g., disease-associated microglia [DAM] and reactive astrocytes [A1]) (Figure 1C and Figure 2). Proinflammatory signaling is then upregulated, harmful cytokine secretion is prominent, and phagocytic-mediated clearance of amyloid-beta and tau is impaired [84,85,86,87,88]. These abnormal states have been extensively characterized at a single-cell resolution and categorized as canonical cellular phenotypes for the known dysfunction of the innate immune system in AD [88,89]. It is speculated that DAM and A1s initiate a neurotoxic positive feedback loop through their consistent cytokine secretion (TNF-α, IL-1β, and IL-6) and complement recruitment (C1q, C3, C5, and C9). This exacerbates the release of additional damage signals and further propagates a cycle of inflammation and neurodegeneration [8,13,90]. These proinflammatory signals can access non-glial immune niches by traveling through CSF-mediated spaces, thereby inducing a weakening of CNS borders and recruitment of CNS lymphocytes [91]. Activated glia further support the infiltration of adaptive immune cells into the AD brain parenchyma and CSF through the production of key lymphocyte-attracting chemokines (CXCL10, CXCL12, CXCL16, and CCL3/5) [13,92,93,94,95,96,97].

## 5. T Cells in the CNS

As AD progresses, there are changes in the adaptive immune cell landscape, most notably the existence of an expanded population of functionally active, antigen-specific CD8 T cells found in the CSF of cognitively impaired patients, but not healthy individuals [98]. Further studies revealed that increased trafficking of these disease-associated T cells was due to elevated chemokine signaling (CXCL10:CXCR3 and CXCL16:CXCR6) from microglia [94,95,96]. The accumulation of CD8 T cells is positively correlated with cognitive dysfunction and, depending on their origin (peripheral vs. CNS) and stage of disease (early vs. late), can either contribute to or mitigate microgliosis, neuroinflammation and neuron degeneration [94,96,99].

CD4 T cells are known to interact with the CNS in homeostasis and in neurodegeneration. In mice, CD4 T cells are required for normal microglial development [100], and Th2-produced IL-4 has been found to be neuroprotective following nerve injury [101]. In mouse models of AD, transferred IFN-γ-producing Th1 cells were capable of increasing plaque burden, microgliosis, and cognitive deficits [102]. Several groups have cataloged changes in abundance of peripheral CD4 T cell subsets in AD patients, including Th17 and Treg cells, suggesting an imbalance in proinflammatory and anti-inflammatory helper T cells. Furthermore, scRNA-seq analysis of intrathecal immune cells revealed that Tregs had a large number of significantly differentially expressed genes in patients with AD compared to healthy aged controls, including increased expression of the lineage defining transcription factor FOXP3 [95]. A significant amount of attention has been paid to Tregs in AD, as multiple studies have found that increasing Treg numbers in preclinical models suppresses disease progression. Others have developed chimeric antigen receptor (CAR) Tregs that recognize amyloid-beta, which were effective in preclinical models. A phase 2a clinical trial evaluating the safety of IL-2 injection for the treatment of AD demonstrated increased numbers of Tregs in the treatment group compared to the placebo, as well as trending positive effects on AD-related biomarkers and the rate of cognitive decline [103]. Taken together, CD4 T cells likely regulate the magnitude of neuroinflammation primarily through the production of proinflammatory and anti-inflammatory cytokines, which exacerbate and blunt disease progression, respectively.

## 6. B Cells in the CNS

The extensive support for T cell involvement in Alzheimer’s disease brings up the question, what about B cells? As stated earlier, characterizations of the skull bone marrow niche and meninges revealed the occupation of CNS-derived developing B cells under healthy conditions [71,77]. Also, through exposure to CNS-specific antigens, these B cells are tested for autoreactivity and subsequently eliminated via negative selection if not tolerized first [71,78]. Certain subtypes of B cells, such as the B1a lineage, even contribute to healthy CNS development through the support of neonatal oligodendrocyte genesis and maturity [66]. However, these normal characteristics are dysregulated under conditions of extreme neuroinflammation, shifting B cell activity towards elevated activation, abnormal parenchymal infiltration, and harmful effector functions [104,105].

One example is multiple sclerosis (MS), a chronic neurodegenerative disease defined by pathogenic de-myelination and neuronal death, which results in elevated meningeal inflammation and autoreactive lymphocyte infiltration into the brain. MS B cells and plasma cells are predominantly antigen-experienced and class-switched; they are a robust population occupying the CSF/subarachnoid space, leptomeninges, and perivascular spaces [106,107]. MS B cells are heavily involved in recruiting and activating T cells, while also adopting their own inflammatory phenotype, leading to clonal expansion, cytokine secretion, and autoantibody production [105,108,109]. In efforts to halt the mobilization of these pathogenic lymphocytes, B cell-targeted drug therapies have been widely successful in mitigating lesions and MS relapse [110,111].

However, few of these characteristics can be extrapolated into AD. A shared key component is the presence of ectopic lymphoid structures, which serve as follicle-like niches that can support the maturation, expansion, class-switching, and persistence of B cells in the CNS [109,112,113]. This was consistent with AD models 5xFAD and APP/PS1, as these lymphocyte-rich immune foci were extensively localized to the dural sinuses in the meninges [114]. Nonetheless, the coincidence may simply be an aging phenomenon that is exacerbated by the inflammatory niches present in MS and AD. Pathophysiological comparisons between the two show that the differences regarding their molecular dysregulations, genetic risk factors, and clinical symptoms present significant hurdles from blindly associating the established B lymphocyte behavior in MS as identical to its potential phenotype in AD [4,7,8,12,14,54,105]. This may offer suggestions towards the mechanisms by which B lymphocytes affect CNS health in neurodegenerative diseases, but further investigation is needed within the disease context itself.

### 6.1. B Cells in Alzheimer’s Disease

B cell involvement in AD is not clearly defined; they may be protective or pathogenic, as summarized in Table 2. A few groups have investigated the role of B cells in AD progression either through transgenic and drug-mediated B cell depletion in mice, or through correlative studies in humans. Total B and T cell ablation in recombination-activating gene (RAG)-deficient mice crossed to the 5xFAD (AD) model resulted in increased disease. This included elevated amyloidosis and microgliosis compared to their immune-competent AD littermates [115]. The exacerbated amyloid pathology was also recapitulated in studies using targeted B cell depletion in AD mice (anti-CD19 and anti-B220) [116]. In AD patients, transcriptomic analysis of peripheral blood mononuclear cells (PBMCs) revealed a negative correlation between the frequency of B cells and clinical dementia scoring [116]. These results suggest that B cells play a protective role in slowing AD pathology. However, other studies of B cell depletion in AD mouse models denoted a counter relationship with AD. Using both drug-targeted (anti-CD20 and anti-B220) and transgenic (J_H_T: 3xTgAD and APP/PS1) B cell knockout constructs resulted in decreased behavioral defects, amyloid plaque formation, and inflammatory microglial activation [117]. Longitudinal studies of PBMCs from a separate clinical cohort of AD patients supported this relationship by revealing a positive correlation between B cell populations and brain amyloid plaque [118]. This conflicting rhetoric regarding the protective or pathogenic nature of B cells in AD implies that they have a more nuanced role that is dependent on several factors.

An explanation for these seemingly opposite results may be due to discrepancies in experimental design. Different AD mouse models (APP/PS1, 5xFAD, and 3xTgAD) were used, with each expressing either purely an amyloid or both an amyloid and tau pathology. As stated in an earlier section, each mouse line consists of genetic constructs that control the onset of the disease; however, the manifestation of their molecular and behavioral phenotypes varies in time and severity (Table 1). This poses an issue with making comparisons between studies, as the manipulations, such as the transgenic breeding or drug treatments, are not consistent. For example, the transgenic depletion of B cells is studied in different models (RAG-5xFAD vs. J_H_T-3xTgAD or J_H_T-APP/PS1), with the conclusions contradicting each other (Table 2). Moreover, while both the RAG and J_H_T lines inhibit the same stage of B cell development (V(D)J recombination), as confirmed by depletion of peripheral B cells, the RAG model also depletes T cells, which may confound some results [115,117]. If both models are accurate, this would suggest that the T cell deletion is more detrimental, masking any beneficial B cell loss.

As for the drug-mediated B cell-deficient groups, the opposing studies varied in terms of which AD model was used (APP/PS1 vs. 3xTgAD or 5xFAD) and subsequently at what age treatment was given (16 weeks vs. 35–47 or 60–70 weeks, respectively). Furthermore, both reports altered how many injections were administered during the regimen of 3 months (21 vs. 3–6 injections, respectively) [116,117]. Therefore, this data is confounded by different stages of the disease (early vs. late), which significantly affects the pathology (amyloidosis, glial activation, lymphocyte infiltration, or cognitive decline). The temporal aspect is a very influential factor, as can be seen with T cell depletion at early- vs. late-stage AD and how it can shift from a protective measure to a detrimental one [99]. Distinct changes occur within the B cell compartment with age [119,120], which may alter the efficiency of CD19/CD20/B220 antibody knockdowns. It has been identified that anti-CD20 treatments only provide a partial knockdown on B1 cells and have no known effect on plasmablasts and plasma cells [121,122,123]. CD19 knockdown may increase the efficiency of B cell depletion by targeting several subtypes, as shown by its depletion of plasmablasts and peritoneal cavity B1 cells to a greater extent [124,125]. However, it is unknown if these treatments are effective on inflammatory B cells (i.e., DN2, CD11c^Hi^, and age-associated B cells). These cells have been shown to contribute to other chronic diseases and may potentially contribute to AD; thus, a more thorough analysis of treatment strategies is required.

An additional unaccounted-for variable in these studies is the lack of sex identification and breeding strategies. Female and male 5xFAD mice develop disease at different rates, with females having more severe plaque burden and neurological deficiency onset earlier than their male counterparts [126,127]. Interestingly, female mice also show increased humoral immune responses [128], a critical avenue of research to pursue in AD pathology. Importantly, the recently identified epigenetic effect in breeding the 5xFAD mouse model [53] suggests that some differences seen in prior studies may be a consequence of breeding rather than treatment. To discern B cell dynamics in the context of AD, there must be consistency within each aspect of the study. It is critical to perform rigorous analyses of several parameters, including the use of multiple transgenic models at various disease states, and a broad spectrum of experimental methods. This will answer whether the opposing results can be attributed to a particular construct, timing, or a yet undiscovered, confounding variable.

A limitation to the current literature is the lack of examination of B cells within the CNS. This is important as B cell populations from different tissues of origin (systemic vs. CNS-derived) influence the humoral response. In aged mice, transcriptomics and clonal analysis revealed antigen-experienced age-associated B cells resident in the dura mater from non-CNS origins [71]. Additional studies in healthy aged and experimental autoimmune encephalomyelitis mice also denoted meningeal occupation of class-switched functional plasma cells from the gut [129,130]. In AD mice, gut-derived plasma cells traffic to the CNS by upregulated CXCR4 expression on B cells. The upregulation is due to increased secretion of chemokine CXCL12 (cognate ligand) from myeloid-derived sources in the brain [97] (Figure 2). This is coupled with an observed reduction in CXCL12 expression in brain endothelial cells and decreased class-switched plasma cells in the AD mouse colon. It is speculated that this migration into the meninges may be facilitated due to a weakening of the blood-CSF barrier; however, further investigation is needed [97].

The CXCL12:CXCR4 chemokine signaling axis and its dysregulation in AD is still relatively novel. In other neurodegenerative contexts (e.g., MS), B cell entry into the CNS is robustly supported by evidence of BBB weakening and elevated CXCL12/13 within the perivascular spaces and CSF [104,105,131]. The observed infiltration from the periphery becomes more pronounced with AD severity; however, this activity by gut-derived B cells has been suggested to be protective for CNS health. Consequently, molecular receptor–ligand interaction analysis reveals that these chemokine-secreting cells in the CNS also upregulate key niche genes necessary for B cell development and residency, including *April*, *Tgfb2/3*, and *Il7* [71,78]. Further investigation is needed to determine whether these interactions are beneficial or detrimental, or both, and if there is a shift in cellular programming that is correlated temporally (early vs. late), as certain molecular aspects of Alzheimer’s appear at different stages of the disease.

### 6.2. Elusive Immunoglobulins

In humans, a significant pathological hallmark to address is the integrity of the BBB. AD is characterized by breakdown and increased permeability of the BBB, contributing to the elevated influx of systemic contents into the CNS, and the subsequent activation of neuroinflammatory and degenerative cascades [132,133,134,135,136,137,138]. In accordance with the activation and migration of B cells from the periphery, autoantibody generation is suspected to occur as an effect of BBB breakage in neurodegenerative diseases [133,139]. Early AD studies show immunoglobulin localization in the parenchyma, as well as the robust presence of antibodies associated with neurofilaments in CSF [140,141]. Since AD is defined by its amyloid pathology, a point of contention is the presence of naturally occurring anti-amyloid antibodies. Studies disagree on whether they are elevated or repressed in disease, and if there is an effect on CNS health [25,28,29,31,142,143,144,145]. As for adaptively generated antibodies, entry into the CNS has been supported by histological analysis of AD mouse brain tissue, which reveals a strong correlation between disease and increased immunoglobulin G (IgG) colocalized with amyloid plaque and microglia [115,117]. While it was speculated that they may target amyloid-beta, quantification of anti-amyloid IgG and amyloid-specific IgG-secreting B cells in the serum and cervical lymph nodes revealed no differences between AD mice and controls [115]. Conversely, other studies showed an increase in amyloid-specific IgG in the serum during AD, both in humans and mice [146,147]. Thus, the questions remain, what is the antigenic target of parenchymal IgG, and are they beneficial or detrimental to AD progression?

## 7. Conclusions

B cells make up less than 5% of lymphocytes in CSF, and very little is known about their function in AD. In mice, evidence suggesting a protective role was shown in several flavors of lymphocyte-deficient mice that exhibited elevated amyloid plaques and neuroinflammation. Conversely, opposite results implying a pathogenic role were found in other B cell-depletion mice that showed reduced AD pathology. Similarly, human studies characterizing the peripheral immune compartment showed, in one instance, a negative correlation with cognitive scoring, yet in another, a positive association with amyloidosis progression. The effector population of the B cell lineage, plasma cells, are a major source of antibody production, and their role in regulating AD severity adds confusion. Infiltration into the CNS from the periphery would imply a dangerous gateway into autoreactivity, as they are not tolerized to the antigens present in this niche. Nonetheless, peripheral class-switched plasma cells were found to patrol the meningeal tissue in normal aged brains. Their recruitment was increased in experimental autoimmune encephalomyelitis and AD mice, as elevated microglial-derived chemokine signaling supported the recruitment of anti-inflammatory plasma cells from the gut.

As for immunoglobulins, inconsistencies regarding the existence and effects of naturally derived anti-amyloid antibodies obscure the lens into understanding humoral immunity in AD. Observations of IgG in the parenchyma of AD mice, alongside activated microglia and amyloid plaques, open a promising avenue for exploration. However, the source of these antibodies and their targets is not clear, and these answers are crucial for clinical research. Drug therapies built on the backbone of anti-amyloid monoclonal antibodies are faced with scrutiny due to limited efficacy with rescuing cognition and potential health risks, especially in individuals with elevated tau pathology [36,37,38,148]. Subsequently a spectrum of clinical efforts targeting tau has been pursued, ranging from controlling its dysregulated post-translational modifications to preventing its aggregation through degradation [148]. Unfortunately, these efforts fall into the same rhythm seen with its amyloid-depleting counterparts. New trials focusing on a combination of the two approaches seem to be promising. Yet, they do not answer the question of what is driving immunoglobulins to migrate to the brain, and what the specificity of these antibodies is. It may be against common neuronal proteins, such as myelin oligodendrocyte glycoprotein, which has been proven to be a source of autoreactivity in B cells during MS [78,104,109]. It could be related to tau pathology, as peripheral IgG infiltration has been observed in transgenic tau mice [13,138]. Also, does the IgG preferentially interact with DAM, which are heavily present in AD, through Fc receptor–immune complex binding? In vitro studies show that co-culturing IgG with human induced pluripotent stem cell-derived microglia exposed to amyloid-beta can either supplement or repress phagocytic capability, depending on the severity of the disease [119]. Investigating this area could potentially elucidate mechanisms to support clinical interventions.

To address these gaps in knowledge, more consistent and thorough experimentation needs to be done. With mouse models, we propose that studies need to move beyond total B cell knockouts, to specifically address the role of B1, B2, plasma cells, and age-associated B cells in disease. These studies should be performed in both sexes of mice with specific breeding strategies (i.e., 5xFAD male breeders). Additionally, the availability to CreERT2/loxP systems coupled with select Cre-backgrounds would boost our understanding of the temporal role of B cells in disease progression. In humans, in-depth studies investigating AD-mediated changes to the humoral immune compartment need to be looked at through CNS-specific sampling (i.e., CSF). Several groups have focused on T cells in CSF from individuals diagnosed with a range of diseases from mild cognitive impairment to AD. The isolation of CNS-associated B cells and plasma cells for single-cell sequencing for RNA and B cell receptors would yield valuable information. B cell receptor analysis would identify clonal expansions and aid in tracking clonotypes that may be disease-associated. This information would provide a strong foundation for the role of B cells in AD, with potential for discovering AD-associated neurodegenerative biomarkers and opening the possibility for increased combinatorial drug treatments.

## Figures and Tables

**Table 1:** Murine models of AD.

Model	Promoter	Construct	Pathology	Molecular & Cellular Phenotype	Cognitive Decline	Reference
PDAPP	PDGF-beta	huAPP: Indiana (V717F)	Increases Ab42 production.	Amyloid-beta deposits start around 6 mo (hippocampus, cortex). Dense plaque formation appears at 9 mo. Elevated gliosis around 6 mo.	Memory defects at 3–6 mo.	Games et al. [43]; Dodart et al. [44]; Hartman et al. [45]
APP/PS1	*Thy1*	huAPP: Swedish (K670N/M671L)	Increases total amyloid-beta production.	Early-onset amyloidosis (Ab42) at 1.5 mo. Initial plaque formation at 3–4 mo (hippocampus). Dense plaque cores by 8 mo. Elevated gliosis at 4 mo.	Memory defects at 7–8 mo.	Radde et al.; [46] Maia et al. [49]
		huPSEN1: (L166P)	Increases Ab42 production.			
5xFAD	*Thy1*	huAPP: Swedish (K670N/M671L)	Increases total amyloid-beta production.	Early-onset amyloidosis (Ab42) around 1.5 mo. Heavy plaque burden by 2 mo (hippocampus, cortex). Elevated gliosis around 2–4 mo. Synaptic dysfunction as early as 4 mo. Severe neurodegeneration detectable at 9 mo.	Memory defects at 3–6 mo.	Oakley et al. [47]; Jawhar et al. [50]
		huAPP: Florida (I716V)	Increases Ab42 production.			
		huAPP: London (V717I)				
		huPSEN1: (M146L)				
		huPSEN1: (L286V)				
3xTgAD	*Thy1*	huAPP: Swedish (K670N/M671L)	Increases total amyloid-beta production.	Early-onset amyloidosis at 3–4 mo. Plaque formation at 6 mo (hippocampus, cortex). Elevated gliosis around 7 mo. Synaptic dysfunction apparent by 6 mo. Tau tangles start to form around 12 mo.	Memory defects at 4 mo.	Oddo et al. [48]; Billings et al. [51]
		huPSEN1: (M146V)	Increases Ab42 production.			
	N/A (knock-in)	MAPT: (P301L)	Promotes tau aggregation/filament synthesis.			
APP-NLGF	N/A (knock-in)	huAPP: Swedish (K670N/M671L)	Increases total amyloid-beta production.	Early-onset amyloidosis at 2 mo. Plaque formation by 2–4 mo (hippocampus, cortex). Elevated gliosis around 9 mo.	Memory defects at 6 mo.	Saito et al. [52]
		huAPP: Iberian (I716F)	Increases Ab42 production.			
		huAPP: Arctic (E693G)	Promotes amyloid-beta aggregation/reduces degradation.			

N/A, not applicable.

**Table 2:** Conflicting roles of B cells in Alzheimer’s disease.

Model System	Manipulation	Experimental Context	Findings	Reference
**Protective**				
Murine	RAG-5xFAD (*Rag2*^−/−^/*Il2rγ*^−/−^: 5xFAD)	Double-knockout (*Rag2*, *Il2rγ*) transgenic mice crossed to 5xFAD strain. B and T lymphocyte knockout.	Deficiency of B and T cells in 5xFAD mice increased Aβ and proinflammatory cytokines (IL-1β, IL-6, TNF-α) in the brain.	Marsh et al. [115]
Human	N/A (PBMCs from AD and normal patients)	PBMC isolation from either healthy or AD individuals (early, late AD).	Negative correlation between peripheral B cells and clinical dementia rating in AD patients.	Xiong et al. [116]
Murine	APP/PS1 + anti-CD19/anti-B220	16-week-old APP/PS1 mice received 21 injections i.p. of anti-B cell monoclonal antibodies over 3.5 months.	B cell depletion in APP/PS1 mice increased amyloid plaque in the cortex and hippocampus and cognitive/memory deficits.	Xiong et al. [116]
**Pathogenic**				
Murine	3xTgAD-J_H_T; APP/PS1-J_H_T (Igh-J^tm1Cgn^/J)	Single-knockout (Immunoglobulin heavy chain J) transgenic mice crossed to either 3xTgAD or APP/PS1 strain. B lymphocyte knockout.	B cell knockouts in AD mice (3xTgAD, APP/PS1) decreased Aβ, inhibited amyloid plaque formation in the cortex, and prevented cognitive decline.	Kim et al. [117]
Murine	3xTgAD; 5xFAD + anti-CD20/anti-B220	60–70-week-old 3xTgAD, or 35–47-week-old 5xFAD, mice received 3–6 injections i.p. of anti-B cell monoclonal antibodies over 2 months.	Drug-targeted depletion of B cells in AD mice (3xTgAD, 5xFAD) reduced hippocampal amyloid plaques and prevented cognitive decline.	Kim et al. [117]
Human	N/A (PBMCs from AD)	PBMC isolation from amyloid-positive individuals (assessed via PiB-PET). Longitudinal analysis.	Positive correlation between peripheral B cells and progression of AD pathology.	Park et al. [118]

N/A, not applicable.

## Data Availability

No new data were created or analyzed in this study.

## Abbreviations

AD	Alzheimer’s disease
APP	amyloid precursor protein
BBB	blood–brain barrier
CNS	central nervous system
CSF	cerebrospinal fluid
DAM	disease-associated microglia
FAD	familial Alzheimer’s disease
MS	multiple sclerosis
PBMC	peripheral blood mononuclear cell
RAG	recombination activating gene
